# Neural Correlates of Vocal Auditory Feedback Processing: Unique Insights from Electrocorticography Recordings in a Human Cochlear Implant User

**DOI:** 10.1523/ENEURO.0181-20.2020

**Published:** 2021-01-15

**Authors:** Charles A. Miller, Roozbeh Behroozmand, Christine P. Etler, Kirill V. Nourski, Richard A. Reale, Hiroyuki Oya, Hiroto Kawasaki, Jeremy D. W. Greenlee

**Affiliations:** 1 Human Brain Research Laboratory, Department of Neurosurgery, The University of Iowa, Iowa City, 52242 IA; 2 Speech Neuroscience Laboratory, Department of Communication Sciences and Disorders, University of South Carolina, Columbia, 29208 SC; 3 Department of Otolaryngology, Head and Neck Surgery, The University of Iowa, Iowa City, 52242 IA; 4 Iowa Neuroscience Institute, The University of Iowa, Iowa City, 52242 IA

**Keywords:** epilepsy surgery, hearing loss, speech production

## Abstract

There is considerable interest in understanding cortical processing and the function of top-down and bottom-up human neural circuits that control speech production. Research efforts to investigate these circuits are aided by analysis of spectro-temporal response characteristics of neural activity recorded by electrocorticography (ECoG). Further, cortical processing may be altered in the case of hearing-impaired cochlear implant (CI) users, as electric excitation of the auditory nerve creates a markedly different neural code for speech compared with that of the functionally intact hearing system. Studies of cortical activity in CI users typically record scalp potentials and are hampered by stimulus artifact contamination and by spatiotemporal filtering imposed by the skull. We present a unique case of a CI user who required direct recordings from the cortical surface using subdural electrodes implanted for epilepsy assessment. Using experimental conditions where the subject vocalized in the presence (CIs ON) or absence (CIs OFF) of auditory feedback, or listened to playback of self-vocalizations without production, we observed ECoG activity primarily in γ (32–70 Hz) and high γ (70–150 Hz) bands at focal regions on the lateral surface of the superior temporal gyrus (STG). High γ band responses differed in their amplitudes across conditions and cortical sites, possibly reflecting different rates of stimulus presentation and differing levels of neural adaptation. STG γ responses to playback and vocalization with auditory feedback were not different from responses to vocalization without feedback, indicating this activity reflects not only auditory, but also attentional, efference-copy, and sensorimotor processing during speech production.

## Significance Statement

This unique study directly examined cortical activity during speech vowel sound vocalization and listening tasks in a deaf subject with bilateral cochlear implants (CIs) and medically intractable epilepsy. Because the subject could not experience bone or air-conducted auditory feedback, unique insights into speech production were made possible. Our findings demonstrate that γ activity in the superior temporal gyrus (STG) reflects a combination of auditory and non-auditory related activation of the auditory cortex during speech production.

## Introduction

Cortical evoked potentials aid studies of brain function and auditory coding provided by cochlear implants (CIs). Electroencephalography (EEG), magnetoencephalography, and functional magnetic resonance imaging have improved brain mapping efforts. Additionally, neurosurgical subjects with medically intractable epilepsy provide unique opportunities to directly record local field potentials (LFPs) through subdural electrodes during perioperative monitoring periods.

Subdural recordings provide clinical benefits, foremost being precise localization of seizure foci for directed tissue resection and mapping of language areas for preservation of speech function (Elger and Burr, 1994; Towle et al., 2008). Parallel research studies also benefit from better localization of neural generators and improved recording quality because of the absence of scalp and bone tissues that spatially and temporally low-pass filter and attenuate neurogenic activity (Pfurtscheller and Cooper, 1975). Compounding the difficulties in studying CI-evoked cortical responses are large electrical artifacts from the CIs themselves that contaminate recordings (Gilley et al., 2006).

A unique case of a subject with bilateral CIs who underwent clinical and research protocols related to epilepsy was described previously (Nourski et al., 2013b), the first report of electrocorticographic (ECoG) responses from a CI user. That report established the feasibility of recording CI-evoked LFPs and described cortical responses to auditory stimuli ranging from click trains to speech. Averaged evoked potentials (AEPs) and event-related band-power (ERBP) spectrograms were reported, with a conclusion that responses were comparable to those of normal-hearing subjects. Here, we focus on cortical responses to self-vocalization tasks in the same subject to investigate patterns of activation within auditory cortex during vocal production.

The subject described here provided a rare opportunity to evaluate possible roles of auditory feedback and other mechanisms (e.g., motor commands or somatosensory feedback), as all auditory feedback could be eliminated by deactivating the subject’s CIs. Our unique investigation is relevant to better understanding sensorimotor interactions subserving vocal production, refinement of vocal control models (Guenther, 2006), and potential development of cortically-based prostheses to restore speech to impaired individuals. This subject’s reliance on CIs for auditory perception eliminated the confounding effect of bone conduction present in normal-hearing subjects; thus, acoustic masking was not needed to block auditory feedback. This is advantageous, as masking sounds also activate auditory cortex and may complicate ECoG or EEG interpretation.

We had a particular interest in modulation of auditory cortical activity by different auditory feedback conditions. It is known that cortical activity in the high γ range (70–150 Hz) to self-vocalization with normal auditory feedback is typically attenuated relative to the condition of listening to played-back vocalization (Houde et al., 2002; Flinker et al., 2010; Behroozmand and Larson, 2011; Greenlee et al., 2011). Conversely, unanticipated experimental auditory feedback alterations during speaking (e.g., level or pitch shifts) alter high γ responses in higher-order auditory (Greenlee et al., 2013) and somatosensory (Chang et al., 2013) cortices such that response attenuation is not typically observed. Likewise, alterations in attention during a listening task can modulate high γ responses in lateral superior temporal gyrus (STG; Ray et al., 2008). Taken together, these findings support the concept of top-down projections modulating auditory cortical function. One such top-down model of corticocortical interaction during speech production is centered on the notion of the “efference copy” mechanisms where motor-related neural activities (i.e., corollary discharges) are postulated to internally predict incoming sensory feedback associated with the intended vocal outputs (Feinberg, 1978; Ford and Mathalon, 2005; Wang et al., 2014). A consequence of this effect is the attenuation of auditory neural responses to normal speech feedback that is predicted by efference copies (Houde et al., 2002; Flinker et al., 2010; Behroozmand and Larson, 2011). It is posited that the efference copies are also implicated in feedback-based monitoring and control of speech production errors (Guenther, 2006), consistent with the hypothesis of increased high γ activity during altered auditory feedback as a possible manifestation of an externally induced error signal (Guenther, 2006; Chang et al., 2013; Greenlee et al., 2013). Top-down and bottom-up circuits may involve cortical activity within different frequency bands, with high γ uniquely involved in top-down control (Fontolan et al., 2014). Indeed, high γ coherence between frontal and auditory cortices during speech production is reported (Kingyon et al., 2015).

Because the current subject presented the opportunity to eliminate auditory feedback during vocalization, the roles of somatosensory feedback and efference copy activity produced during vocalization could be explored independent of activating the auditory system. Previous studies have demonstrated that auditory cortex receives somatosensory input (Tremblay et al., 2003; Tourville et al., 2008; Golfinopoulos et al., 2011; Lametti et al., 2012). As movement of the orolaryngeal apparatus occurs during vocal production, ECoG responses in the absence of auditory feedback might reflect somatosensory bottom-up mechanisms. This, in part, motivated our study.

Finally, we sought to demonstrate how signal processing can reduce electrical artifacts in neural recordings obtained during CI stimulation. CI artifacts interfere with scalp-based EEG measures, leading to various approaches to their amelioration (Gilley et al., 2006; Martin, 2007; Friesen and Picton, 2010; Sinkiewicz et al., 2014). We examined the utility of the spline-Laplacian transform, which has had wide application to scalp potential studies, and noted how the transform influenced our ECoG data analyses.

## Materials and Methods

### Subject

The subject was a 58-year-old female with bilateral hearing loss induced by ototoxic antibiotic treatment at age 38. Pure-tone thresholds were at or worse than 90-dB hearing loss bilaterally, with 0% correct scores on spondee, Northwestern University Auditory test six words (Tillman and Carhart, 1966), and Central Institute of the Deaf sentences (Hirsh et al., 1952). Her right cochlea was implanted with an eight-channel Clarion C1 prosthesis at age 39 and an Advanced Bionics HiRes 90K implant in her left cochlea at age 53. The subject had used stable CI settings for months before this study. Specifically, the right CI used an Advanced Bionics Harmony processor with monopolar continuous-interleaved sampling (406 pulses/s per channel, 150-μs/phase biphasic pulses). As electrode three was defective, seven of eight intracochlear electrodes were used. The left CI used an Advanced Bionics Auria BTE processor and 14-channel HiRes-P program (3458 pulses/s per channel, 21-μs/phase biphasic pulses). At the time of data collection, her consonant-nucleus-consonant (monosyllabic) word scores were as follows. For the right ear, she achieved %-correct phoneme and word scores of 66 and 39, respectively, for the left ear scores were 60 and 31, and with bilateral stimulation, scores were 73 and 51. With her implants off, the subject reported an inability to hear any sound, including her own voice, and did not report any sensation or vibration in her head during vocalization.

After her hearing loss, the subject developed medically intractable epilepsy and underwent a 3-d period of temporary implantation of subdural recording electrodes positioned over the temporal lobe to localize seizure foci before definitive neurosurgical treatment. This electrode array was clinically necessary, as non-invasive evaluation of the subject’s epilepsy suggested a seizure focus in the left temporal lobe but did not precisely locate a site suitable for resection. Preoperative amobarbital Wada testing (Geschwind, 1970) confirmed left cerebral dominance for language.

The inpatient video-ECoG monitoring confirmed a seizure focus within the mesial left temporal lobe; this focus did not involve any of the cortical sites activated by CI stimulation (Nourski et al., 2013b). The subject had no other significant medical conditions and she gave written informed consent for participation in this study, including publication of results. Study protocols and consent were approved by the University of Iowa Institutional Review Board in compliance with federal regulations and the principles expressed in the Declaration of Helsinki.

### Study design and experimental stimuli

We focused on examining γ (32–70 Hz) and high γ (70–150 Hz) LFPs recorded from the lateral surface of the temporal lobe, with emphasis on the posterior STG as a region implicated in speech perception. The choice of these frequency bands was motivated by previous research demonstrating their task-related and attention-related modulations (Crone et al., 2011; Nourski and Howard, 2015; Nourski, 2017). Accordingly, we hypothesized that modulation of γ and high γ responses would differ during vocalization with (CIs ON) versus without (CIs OFF) auditory feedback conditions, with the possibility that the latter may reveal responses mediated by somatosensory feedback and/or efference copies otherwise obscured by dominant auditory feedback. We also examined the use of the spline-Laplacian transformation (Nunez and Pilgreen, 1991; Nunez et al., 1994) to reduce CI-induced artifacts. This method is appropriate for minimizing CI artifacts, as they presumably are common to many recording channels. Based on the second spatial derivative of LFPs, the spline-Laplacian attenuates common-mode signals and “sharpens” or produces more spatially localized LFPs at the expense of distant, volume-conducted potentials. We also examined very high γ (156–300 Hz) responses, as our recording system acquired potentials at a sampling rate sufficient to explore this frequency band.

We sought to observe how AEPs from the STG were modulated under different vocalization and feedback conditions through four experiments. These were based on hypothesized presence or absence of auditory system feedback, somatosensory feedback, and the efference copy. Under all conditions, the speech sound was a sustained vowel phonation/a/continued at a constant pitch, produced either by the subject during each trial or played back to her from recordings made during previous vocalization trials. To replicate the conditions that the subject used in everyday listening and vocal production, both left and right CIs were active and at her optimized, presurgical settings.

In the first experiment, the subject vocalized at a self-guided pace and auditory feedback was provided through the CIs, using their internal microphones (“vocalization-microphone feedback” condition). In the second, the subject again vocalized, but auditory feedback was provided by an external microphone whose output was routed to the two CI auxiliary input jacks (“vocalization-Aux In feedback”). This condition was used as a control to facilitate comparisons with the fourth (playback) condition where the input jacks were used to deliver recorded utterances back to the subject. The third condition also required vocalization, but both CIs were turned off, depriving the subject from auditory feedback (“vocalization-no auditory feedback”). Finally, the fourth condition was studied in which the subject did not vocalize, but a recording of her previous vocalizations was played back using the CI auxiliary inputs (“no vocalization-playback via Aux In”). Each of these previous vocalizations was obtained from those recorded during the “vocalization-Aux In” condition so that the subject heard each of those 40 unique vocalizations as each playback trial. She was instructed to listen to the recording. Table 1 outlines the four experimental conditions.

**Table 1:** Summary of the four experimental conditions with notations of the presence or absence of auditory feedback, somatosensory feedback, and presumed vocal-motor efference copy

Task and auditory stimuli	Auditory feedback?	Somatosensory feedback?	Efference copy?
Vocalization: microphone feedback	Yes	Yes	Yes
Vocalization: Aux In feedback	Yes	Yes	Yes
Vocalization: no auditory feedback (CIs off)	No	Yes	Yes
No vocalization: playback via Aux In	Yes	No	No

For the three vocalization conditions, the subject was instructed to repeatedly phonate for approximately a 1-s duration at a self-determined pace and to maintain a consistent loudness, using her normal conversational level. As her everyday CI settings were not manipulated, this could result in different vocal efforts across experiments, since feedback was either eliminated, provided by the built-in CI microphones, or provided through our microphone with its signal routed to the CI Aux In port. Note that because of the physical connecting to and activation/deactivation of the CIs, it was not possible to interleave the four experimental conditions and they were performed sequentially as listed in Table 1.

### Electrophysiological recording

LFPs were recorded for clinical and research purposes using a 96-contact array of platinum/iridium electrode disks embedded in a flat, flexible, SILASTIC carrier (AdTech). Its 2.3-mm diameter disks had a 5-mm center-to-center spacing and were arranged in a uniform 8 × 12 grid layout of contacts, providing a rectangular area of coverage of 3.5 × 5.5 cm, measured from electrode centers. Localization of this array over the left temporal lobe was determined through registration of preimplantation and postimplantation CT scans, as well as intraoperative photography (Nourski et al., 2013b). Electrode coordinates were then mapped onto a template brain (ICBM152 average, Montreal Neurologic Institute) to obtain a 3-D surface rendering of all recording sites as well as the ipsilateral CI receiver package.

Stimulus delivery and response recordings were controlled by a TDT RZ2 multichannel real-time processor (Tucker-Davis Technologies). All LFPs were recorded in monopolar fashion using single electrodes of the array and a subgaleal reference electrode placed under the scalp in the left posterior frontal area. LFPs were first low-pass filtered by a TDT PZ2 unity-gain preamplifier module (0.35 Hz to 7.5 kHz 3-dB frequencies, 24-dB/octave filter slopes) and digitized at 24,414 samples/s with 18-bit resolution. This representation was downsampled by the TDT RZ2 BioAmp Processor at 2034 samples/s for more efficient storage and processing of large datasets. During downsampling, bandpass filtering was performed by a TDT RZ2 digital filter with 0.7- to 800-Hz cutoff frequencies.

The output of a Shure Beta 87C electret microphone (Shure) was used to record all vocalizations. Its output was amplified (10 dB) by an UltraLite mk3 Hybrid sound processor (MOTU) before being sampled by a TDT RP2.1 Real-Time Processor which low-pass filtered the microphone output at 5127 Hz (3-dB cutoff) and digitized at 12,207 samples/s at 24-bit resolution. Digitized LFPs and microphone signals were stored to disk for *post hoc* analysis. We sought to collect 40 repeated trials in each of the four conditions; as noted below, fewer trials were obtained in some conditions.

### Data analysis and transformations

All ECoG and voice waveforms were analyzed using custom MATLAB scripts (MathWorks). The following denoising algorithms were used to remove large transients and narrowband noise (such as line noise) in the ECoG recordings. Transients were identified by iteratively transforming voltages to *z* scores and discarding values greater than *z *=* *10 until no further outliers remained; visual inspection of all trials was used for confirmation. Trials with outliers were removed from the dataset before averaging and not analyzed. This rejection served to remove artifacts created by interictal spiking or muscle activity. Narrowband line noise was reduced using the complex demodulation approach (Papp and Ktonas, 1977; Ktonas and Papp, 1980; Kovach and Gander, 2016).

Voice onset times were first determined for each trial by threshold detection of audio waveform amplitude exceeding noise background levels and then confirmed/refined via visual inspection of onset of each utterance. Trials with voice waveforms found to contain artifacts were discarded. Vocalizations were assessed for duration, interutterance interval, level as recorded by our microphone, and voice fundamental frequency (*F*_0_) as derived by Praat speech analysis software (http://www.praat.org; Boersma, 2001). Voice onset times were used to define the onset of analysis windows so that event-related across-trial AEPs and ERBP spectrograms could be computed (Greenlee et al., 2011, 2013).

ERBP spectrograms were calculated by using a preutterance, 200-ms silent period (500–300 ms before voice onset) to which the postvoice onset amplitude data within each frequency band were normalized. The energy in each frequency band was derived using Morlet wavelet decomposition (Oya et al., 2002) for frequencies ranging from 2 to 300 Hz, yielding a total of 75 3.973-Hz-wide analysis bins. Band-specific responses were derived from ERBPs by averaging the power across contiguous bins for each of the three γ bands (i.e., γ, high γ, and very high γ with frequency ranges defined above). For graphical portrayal in this report, ERBP-derived band time waveforms were smoothed using a 50-ms rectangular moving average filter so that the envelopes were emphasized; however unsmoothed waveforms were used for statistical analyses.

The LFP is dominated by the electric activity of neurons in restricted regions beneath each recording electrode; however, they may also contain volume-conducted common-mode activity from distant sources. In the case of CI stimulation, LFP recordings will also likely be contaminated by CI-generated electric artifacts, as they are in scalp potential recordings. Thus, we examined the efficacy of applying a two-dimensional version of the spline-Laplacian transformation (Nunez and Pilgreen, 1991; Nunez et al., 1994) to the recorded LFP waveforms (i.e., 20-Hz lowpass filtered). The spline and the subsequent Laplacian used here are analytical solutions, rather than numerical approximations, to the second-order spatial derivatives required for the computation. This technique performs high-pass spatial filtering based on the second spatial derivative across the two-dimensional array of recording sites, reducing common-mode signals and emphasizing local activity. The spline-Laplacian is commonly used and has been previously used with ECoG data (Reale et al., 2007; Nourski et al., 2013a).

### Statistical analyses

Statistical significance of differences observed in the assessments of vocalization parameters were determined using non-parametric Wilcoxon rank-sum tests, corrected for multiple comparisons using the false discovery rate approach (Benjamini and Hochberg, 1995). Evaluation of the ECoG responses focused primarily on the mean power in the γ, high γ, and very high γ bands. Mean power resulting from vocalizations or playback of vocalizations were assessed over a 400-ms window that began at the onset of vocalization or playback of vocalization. These mean power values were referenced to the mean power computed during a 290-ms analysis window occurring 390–100 ms before vocalization or playback onset. The preonset window was selected to fall in a “silent” period and avoid contamination from any preceding utterance and allow for identification of any activity occurring before vocalization. The postonset window was chosen to cover the time period of maximal high-frequency response amplitude. As we examined ECoG band power across two analysis time windows, four largest amplitude recording sites, and four experimental conditions, ANOVA was required. It was conducted using least square errors to fit general linear models, as implemented by the GLM procedure of SAS (SAS Institute), as its flexibility permits non-homogeneity of variance and unequal sample sizes.

## Results

### Vocalization characteristics

**Figure 1.**
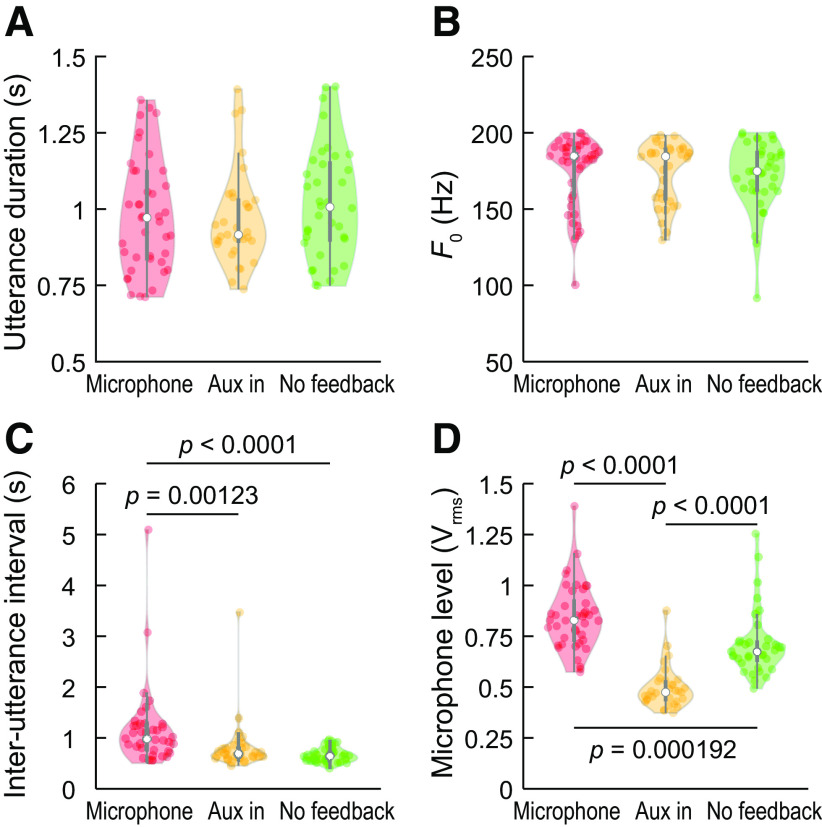
Comparison of voice measures for the three vocal production conditions. ***A***, Utterance duration did not differ across feedback conditions. ***B***, Voice *F*_0_ was slightly lower and more variable when subject vocalized without auditory feedback. ***C***, Intervals between successive self-paced utterances were longer during the microphone feedback condition compared with the no auditory feedback condition. ***D***, Sound level as captured by the recording microphone (*V*_rms_) varied as a function of feedback condition and was greatest when during the microphone condition, and least when the Aux In port was used to present auditory feedback during vocalization. In each violin plot, colored circles represent individual trials, white circle denotes the median, thick gray bar denotes *Q*_1_ and *Q*_3_, and whiskers show the range of lower and higher adjacent values (i.e., values within 1.5 interquartile ranges below *Q*_1_ or above *Q*_3_, respectively); *p* values indicate results of Wilcoxon rank-sum tests, FDR corrected for multiple comparisons.

Across the four conditions (“vocalization-microphone feedback,” “vocalization-Aux In feedback,” “vocalization-no auditory feedback,” and “no vocalization-playback via Aux In”), we obtained 41, 33, 36, and 33 individual trials, respectively. Given the inherent variability in human vocalization and the four feedback conditions investigated, we examined the subject’s vocal production as summarized in Figure 1. Although there were four distinct experimental conditions, the same vocalizations obtained in the “vocalization-Aux In feedback” condition were played back in the “no vocalization-playback via Aux In” experiment; thus, three vocalization datasets are shown in Figure 1.

Vocalization duration and voice *F_0_* did not vary in a statistically significant way across the three vocalization conditions (Fig. 1*A*,*B*), indicating that the subject maintained consistent duration across the study. Across all utterances, median vocalization duration was 956 ms, median voice *F*_0_ was 183 Hz. The interutterance intervals (Fig. 1*C*) were computed as the time since the end of the previous vocalization to the start of the subsequent self-paced vocalization. Interutterance intervals in the “vocalization-microphone feedback” condition had a median of median of 983 ms (Q_1_ – Q_3_ = 749–1257 ms) and were significantly longer compared with those in the “vocalization-playback via Aux In” condition (median 693 ms; Q_1_ – Q_3_ = 620–818 ms, *W *=* *1767, *p *<* *0.0001) and the “vocalization-no auditory feedback” experiment (median 645 ms; Q_1_ – Q_3_ = 558–731 ms, *W *=* *1983, *p *<* *0.0001). Across-experiment changes were observed in the recorded vocalization amplitude, as defined by the microphone level (Fig. 1*D*). Microphone level (*V*_rms_) varied significantly across all three vocalization conditions. “Vocalization-microphone feedback” had greater output levels than both “vocalization-Aux In feedback” (*W *=* *2171, *p *<* *0.0001) and “vocalization-no auditory feedback” (*W *=* *655 *p *= 0.000192) conditions. Measured vocalization amplitude was significantly lower in the “vocalization-Aux In feedback” experiment compared with “vocalization-no auditory feedback” condition (*W *=* *1991, *p *<* *0.0001). In summary, notable across-experiment changes in vocal production as a function of auditory feedback condition were observed as a decrease in voice *F*_0_ when auditory feedback was absent, interutterance duration progressively shortened over the course of the experimental session, and changes in microphone output level across the three feedback conditions did not demonstrate an obvious physiologic pattern.

### AEPs, frequency-time spectrograms, and spline-Laplacian transform

**Figure 2.**
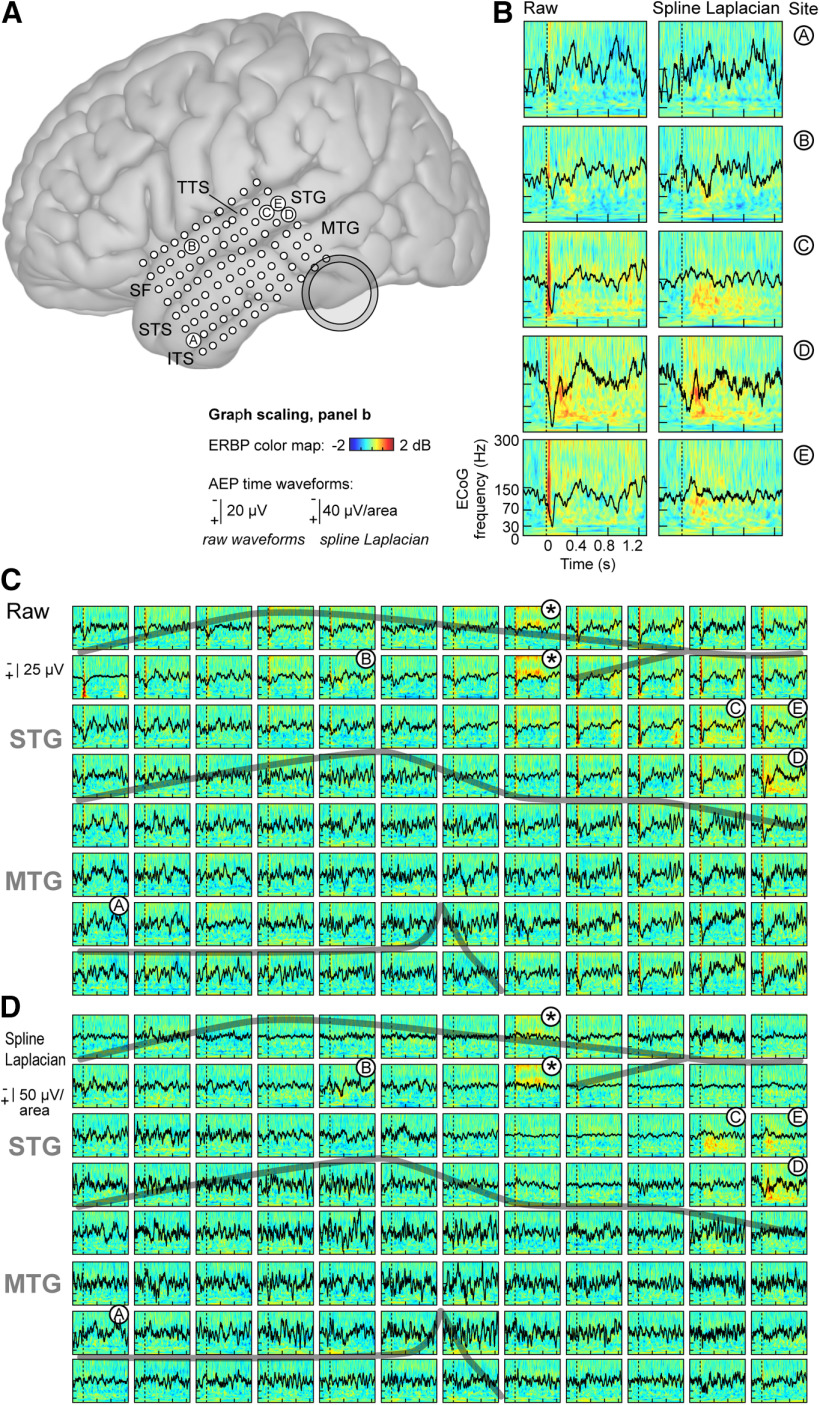
Location of electrode array on brain surface and representative response plots. ***A***, Estimated recording locations of the 96-electrode array as projected onto a template brain surface rendering. Shaded ring indicates the approximate position of the ipsilateral CI receiver. SF: Sylvian fissure; TTS: transverse temporal sulcus, STG: superior temporal gyrus; STS: superior temporal sulcus; MTG: middle temporal gyrus; ITS: inferior temporal sulcus. ***B***, Exemplary data (raw and spline-Laplacian-transformed AEPs and ERBPs) from five recording sites (A–E) are shown for vocalization with auditory feedback via Aux In condition. ***C***, ***D***, Comparison of raw (***C***) and spline-Laplacian-transformed (***D***) AEP (black lines) and ERBP (color plots) data for all 96 recording sites for the vocalization with auditory feedback via Aux In condition. Note the very brief, high-amplitude, broadband ERBP response felt to be CI artifact occurring at the time of voice onset (dashed lines) in the raw data that are eliminated by the transform. The artifact is seen at numerous sites but clusters mainly in the superior-posterior quadrant of the array; prominent artifact is also noted at more anterior, non-contiguous sites. Note a different, longer duration high-frequency response at the two sites marked with asterisks; this is also felt to be CI artifact and is not significantly affected by the transform. The transform adds noise to the AEPs for ∼3/4 of the sites but effectively removes the positive component beginning at time 0 in the posterior-most contacts. Gray lines indicate major sulci. Axes in ***C***, ***D*** are scaled as they are in ***B***.

We observed a prominent brief, transient component in the AEP and ERBP responses on STG (Fig. 2*A*,*B*) when the CI Aux In ports were used for stimulation. Given the unphysiologic temporal properties of these responses in comparison to previous STG responses to self-vocalization in normal-hearing subjects (Flinker et al., 2010; Greenlee et al., 2011, 2013; Chang et al., 2013), we interpreted them as contaminated with CI-related artifacts. AEP time waveforms and ERBP time-frequency plots, selected on the basis of sites with significant activity compared with baseline as visualized in the broadband time-frequency plots, are shown in Figure 2*B* for the “vocalization-Aux In feedback” condition for both unprocessed and spline-Laplacian-transformed data. This experimental condition was chosen for display, as in contrast to the “vocalization-microphone feedback” condition, it demonstrated high-amplitude transients and broadband responses shortly after vocalization onset that were considered artifactual.

These artifacts are notable in two ways. First, they were not uniformly distributed across the 96 recording sites (Fig. 2*C*), and second, they did not follow a simple pattern of volume conduction in which one might expect an intensity gradient based on the posterior-inferior location of the CI receiver relative to the recording array (Fig. 2*A*). While the strongest artifacts clustered in the superior-posterior quadrant of the recording array, several sites in the superior-anterior corner of the array, farthest from the CI receiver, demonstrated prominent artifacts as well (Fig. 2*C*).

Application of the spline-Laplacian transform was effective in reducing CI-induced electrical artifacts superimposed on neural responses. As shown in Figure 2*C*, many recording sites exhibited large amplitude, broad band, and very short duration ERBP increases at vocalization onset. This type of artifact was effectively attenuated by the transform (Fig. 2*B*,*D*). This is consistent with the algorithm’s tendency to reduce common-mode signals, as this “onset” artifact occurred (as judged by visual inspection) in about one-third of the 96 recording sites. However, using the transform also increased noise in the AEPs for many recording sites, as can be seen by comparing the AEPs before and after the transform for the sites in the lower half of the recording array (Fig. 2*C*,*D*).

At two recording sites, we identified sustained high-frequency (>150 Hz) ERBP increases that persisted for the duration of vocalizations (Fig. 2*C*,*D*, asterisks). This pattern is atypical of self-vocalization neurophysiological responses reported in other studies in that its time course is not characteristic of the time-decaying high γ response from lateral STG (Flinker et al., 2010; Greenlee et al., 2011). This very focal ERBP increase was not appreciably altered by the spline-Laplacian, due likely to the very focal nature (i.e., two of 96 contacts) and the insensitivity of the spline-Laplacian to non-common-mode components.

**Figure 3.**
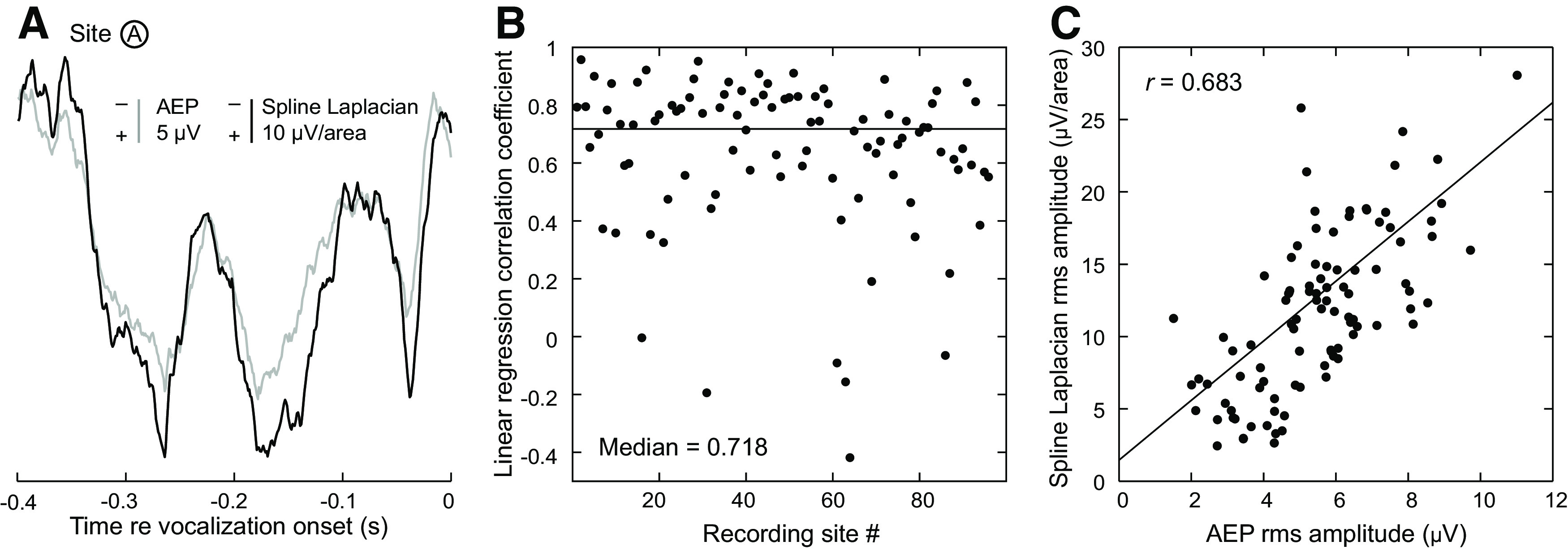
The effects of the spline-Laplacian transform on AEPs. ***A***, Raw and spline-Laplacian-transformed AEPs recorded from site A (Fig. 2) during silent period before voice onset are plotted in gray and black, respectively. ***B***, Correlation coefficients between raw and spline-Laplacian-transformed AEPs for all 96 recording sites, with a median value of 0.718. ***C***, Correlation between raw and transformed rms AEP amplitudes for all 96 recording sites.

We quantified AEPs before and after transformation to characterize how the spline-Laplacian altered AEPs, using the time epoch 0.4–0.1 s before vocalization onset to avoid event-related neural activity. Figure 3*A* shows the overall preservation of the waveform morphology and latency between raw AEP and spline-Laplacian-transformed time waveforms from a recording site with no obvious evoked response (site A; see also Fig. 2). Figure 3*A* also demonstrates characteristic amplitude increases at this site in the transformed waveforms (note different scale bars). Linear regression between these two waveforms (i.e., raw vs spline) revealed strong correlation (*r *=* *0.957) that was highly significant (*t *=* *65.9, *p*_error_ < 0.0001). In this case, 92% of the variance in the spline-Laplacian-transformed waveform is explained by the raw waveform amplitudes.

As with recording site A, good correlations between raw and spline-Laplacian-transformed AEP waveforms were identified for all 96 recording sites. Figure 3*B* shows linear regression correlation coefficients for each of the 96 sites, with a median coefficient of 0.718. Finally, we examined the relationship between the raw and spline-Laplacian-transformed waveforms in terms of the rms value of their amplitudes. Figure 3*C* demonstrates the relationship between spline-Laplacian rms amplitude and raw rms amplitude was correlated (*r *=* *0.683; *t *=* *18.6 and *p*_error_ < 0.0001). After these analyses to validate the fidelity of the spline-Laplacian-transformed data, subsequent analyses were performed on the transformed AEP and ERBP data.

### Hypothesis evaluation

**Figure 4.**
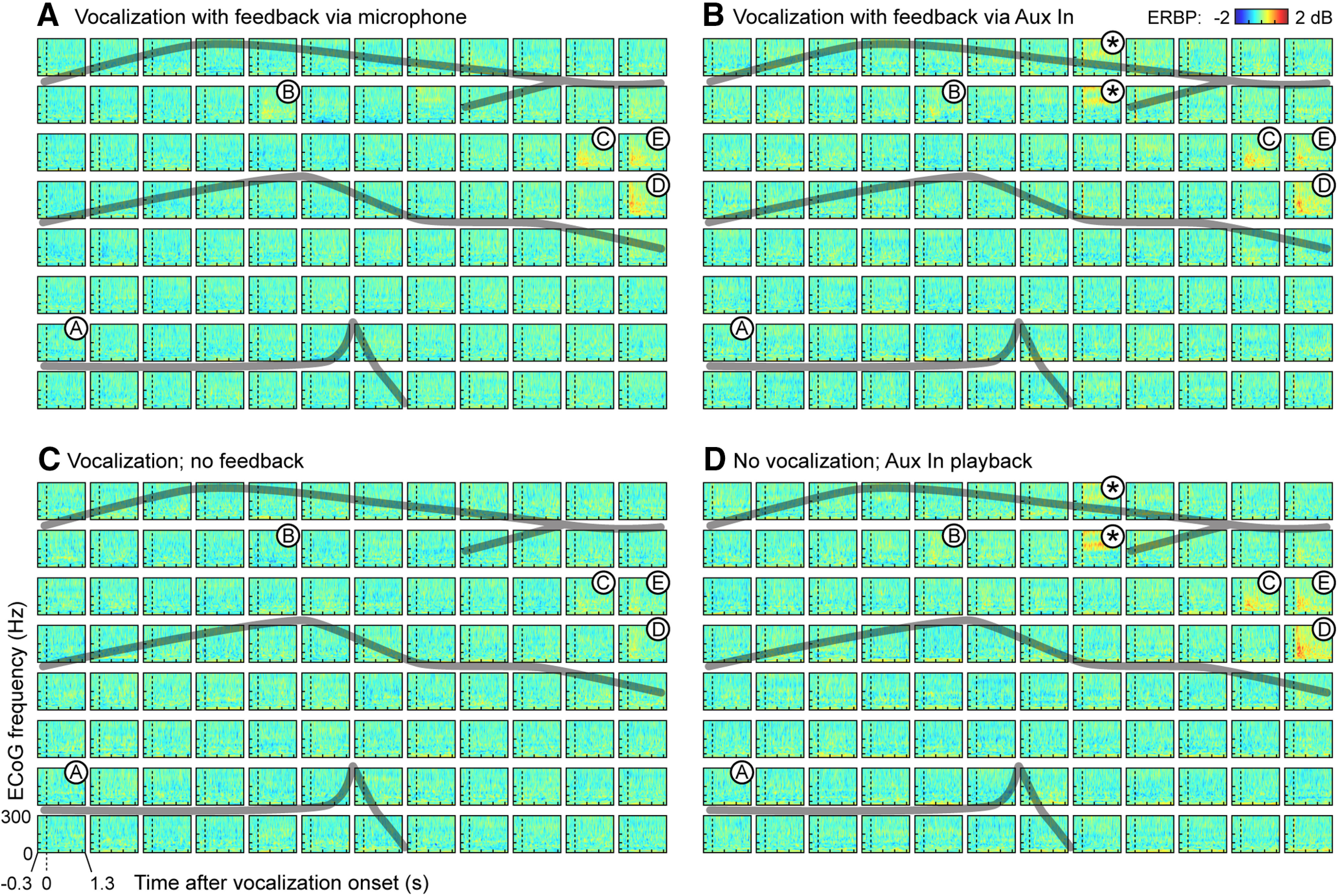
Spline-Laplacian-transformed ERBP results for vocalization with feedback via (***A***) microphone and (***B***) Aux In port, (***C***) without feedback, and (***D***) playback [no vocalization] experimental conditions. Largest-amplitude responses clustered in sites C, D, and E on the posterior STG for all four conditions. Site B also showed responses more anteriorly on STG. Dashed black lines denote voice onset. Gray lines denote major sulci (see also Fig. 2*A*). Asterisks indicate channels with persistent CI artifact in the two conditions where the CI’s Aux In port was used to present auditory stimuli (***B***, ***D***). Spline-Laplacian was not effective in removing this artifact.

**Figure 5.**
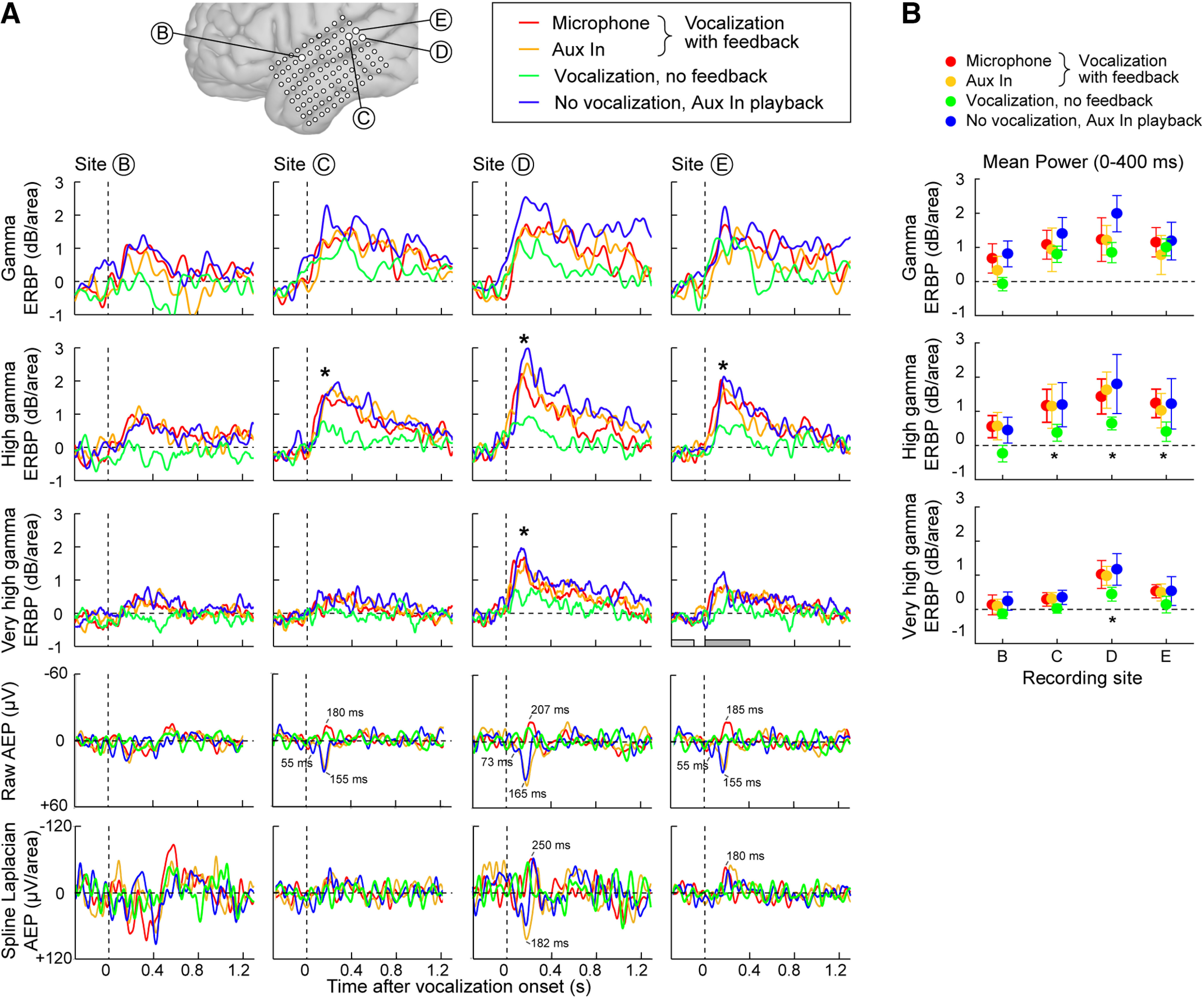
***A***, Band-specific spline-Laplacian-transformed mean ERBP [γ (top row), high γ (second row), and very high γ (third row)] for responsive STG sites B–E (columns) in each experimental condition. Time windows used to statistically compare mean power before and after voice onset are shown as light and dark gray bars, respectively (see site E in third row). Raw AEPs (fourth row) and spline-Laplacian-transformed AEPs (bottom row) are shown for each and each condition. Peak latencies of prominent peaks are noted in ms. ***B***, Plots of mean band-specific power for each recording site and condition. Significant differences in the poststimulus window between mean power of the vocalization with no feedback condition (green plots) and the three other conditions were observed and are indicated by asterisks (ANOVA, *p *<* *0.05).

ERBP results for all four experimental conditions are shown in Figure 4. In the three conditions in which the subject received auditory feedback (Fig. 4*A*,*B*,*D*), responses were evident in a limited number of sites clustered on the posterior portion of the STG (recording sites C, D, and E) and a single recording site (B) on the middle STG. Responses at these sites were predominantly in the γ and high γ bands. Because the subject provided a condition without any auditory feedback, unique insights into the roles of γ and high γ STG responses can be gained. To test our hypothesis that high γ responses would reflect feedback conditions, we examined these responses in detail from the four recording sites that yielded the largest magnitude responses to the vocalizations (sites B–E; Figs. 2, 4). We sought to complement the ERBP response characteristics evident in the time-frequency spectral plots (Fig. 2) by converting the responses to mean power-versus-time waveforms for the γ, high γ, and very high γ bands for the four sites and four experimental conditions (Fig. 5).

All four sites exhibited comparable degrees of γ response amplitude across the four experimental conditions (Fig. 5, top row) and no statistical differences were found by ANOVA. This suggests that the γ band responses from higher-order auditory cortex of the STG are not because of efference copy (which would be absent in playback), somatosensory feedback (absent in playback), or auditory feedback (absent when CIs were off) mechanisms. Notably, all of these lateral STG sites, three of which are located in the posterior STG (sites C, D, and E) are higher-order auditory regions, as confirmed by this subject’s responses to a variety of acoustic stimuli as reported previously (Nourski et al., 2013b). That report showed in particular that site D responded robustly to both basic auditory stimuli (click trains) and speech stimuli.

In the case of high γ power, the responses across sites did differ by feedback condition (Fig. 5*A*,*B*, second row). Specifically, high γ response amplitudes at sites C, D, and E were significantly attenuated during the “vocalization-no feedback” condition (Fig. 5A,*B*, second row, green waveforms) compared with the other three conditions. Importantly, high γ amplitude did not differ between the three conditions with auditory feedback, including both vocalization and playback tasks. The very high γ response amplitudes were only significantly lower in the “vocalization-no feedback” condition at one site (Fig. 5*A*,*B*, third row, site D).

To test these observations regarding different high γ responses, a 2 × 4 factorial ANOVA was used to determine the effects of experimental condition and recording. Our metric of interest was the mean ERBP as determined in the postvoice (and postplayback) onset interval spanning 0–400 ms (Fig. 5*B*), normalized to the mean power in the prevocalization silent interval (i.e., 390–100 ms before onset). Experimental condition was treated as two groups, one being the three conditions in which the subject received feedback or playback and the second being the one without auditory feedback. Four recording sites (B through E) were chosen. The main effects of experimental condition (*F*_(1,539)_ = 68.04, *p*_error_ < 0.0001) and recording site (*F*_(3,539)_ = 14.88, *p*_error_ < 0.0001) were statistically significant. The interaction factor of experimental condition and recording sites failed to reach significance.

The interpretation of the effect of experimental condition is straightforward; ECoG (spline-Laplacian transformed) high γ responses with auditory feedback (either during vocalization or playback) had significantly larger amplitudes than the responses evoked by vocalizations without auditory feedback. The main effect of recording site occurs because the experimental condition effect is significant (*p*_error_ < 0.0001) for all but one site (site B) as assessed by Tukey–Kramer adjustment for multiple pairwise comparisons. As the datasets had unequal sample sizes, we calculated least-squares adjusted means for experimental condition levels and 95% confidence limits (α = 0.01) for each adjusted mean and each difference in adjusted means. The smaller high γ amplitude for the no feedback condition excluded zero values (i.e., response was present) except for recording site B, which had smaller amplitudes overall in comparison to the site C, D, and E responses. Consistent with the literature (for review, see Crone et al., 2006), the high γ responses appear to decay (i.e., return to baseline) at a faster rate than do the γ responses.

In the very high γ band, responses are evident for site D but are smaller at the other sites. A 2 × 4 factorial ANOVA was used to assess the effects of experimental condition (feedback or playback vs no feedback) and recording site (B through E) on the very high γ band responses. Both main effects of experimental condition (*F*_(1,540)_ = 22.0, *p*_error_ < 0.0001) and recording site (*F*_(3,540)_ = 17.3, *p*_error_ < 0 0.0001) were statistically significant, although their interaction factor was not significant. Single degree-of-freedom tests indicated that the experimental condition effect for very high γ ERBP responses to the feedback/playback conditions had significantly larger values than those evoked during the no feedback condition only at recording site D (*F*_(1,540)_ = 17.1, *p*_error_ < 0.0001). The 95% confidence limits (α = 0.01) for adjusted means showed that the smaller very high γ amplitude for the no-feedback condition excluded zero values only for site D, with a mean of 1.0 dB and confidence intervals of 0.48 and 1.6 dB. Thus, vocalization-related changes in ECoG extend to the very high γ band.

Raw (Fig. 5*A*, fourth row) and spline-transformed (Fig. 5*A*, bottom row) AEPs were compared in detail for the same largest ERBP amplitude contacts B–E for the four experimental conditions. The largest peak AEP responses occurred with postvoice onset latencies of 155–250 ms across the four conditions with a smaller earlier peak observed 55–73 ms in the two Aux In conditions (Fig. 5*A*, fourth and bottom rows, blue and orange waveforms). Two observations are noteworthy. First, responses obtained during use of the Aux In port showed reversed polarity and slight prolongation of the largest peak (Fig. 5*A*, fourth row, blue and orange compared with green, red waveforms). Second, the vocalization no feedback condition had notably attenuated AEP peaks.

## Discussion

Results of ECoG recordings in our deaf CI user provide unique insights into the neural mechanisms of vocal auditory feedback processing. Our main finding was that significant γ responses, and to a lesser extent high γ and very high γ, were observed in higher-order auditory cortex on STG in the absence of auditory feedback. We have also demonstrated the utility of the spline-Laplacian transform in removal of CI artifact contamination of neural responses.

### Auditory cortical γ and high γ responses in the absence of auditory feedback

Non-primary regions of the temporal lobe auditory cortex in the posterior STG can demonstrate highly focal responses to speech sounds (Crone et al., 2011; Greenlee et al., 2011; Mesgarani et al., 2014). Responses produced during speech and perception from normal hearing subjects originate from primary and non-primary posterior STG auditory cortical sites (Trautner et al., 2006; Steinschneider et al., 2011; Greenlee et al., 2014). Studies have confirmed the focal nature of these STG responses to various types of sound stimuli (Howard et al., 2000; Brugge et al., 2008; Steinschneider et al., 2011). Our profoundly hearing-impaired CI user demonstrated ECoG responses to speech and non-speech sounds consistent with normal hearing subjects (Nourski et al., 2013b). While this is not unexpected, the nature of the stimulus provided by a CI is substantially different from in the case of acoustic stimulation of the normal ear (Kiang, 1965; van den Honert and Stypulkowski, 1986). Our new data from this CI user further show STG responses comparable overall to those found in normal-hearing subjects specific to vocalization.

We observed two novel findings in the STG γ and high γ responses across the auditory feedback and no feedback conditions. First, we found that STG higher-order auditory cortex γ and high γ responses were observed not only during conditions in which the subject received auditory feedback, but also when auditory feedback was absent (with the CIs turned off). This suggests that STG γ and high γ band responses do not only reflect auditory feedback, efference copy, or somatosensory coding mechanisms, as each one of these mechanisms was absent in at least one of the four experimental conditions (Table 1). We observed that for the STG γ responses specifically, the band-specific power increased during the no-feedback condition and was not significantly different in amplitude than all other experimental conditions, consistent with our hypothesis that this cortical region responds in a multi-modal fashion. For example, this region’s responsiveness is already known to be modified by visual stimuli related to speech production (Reale et al., 2007).

### Potential additional influences on the STG responses

Another significant finding was a diminution in high γ and very high γ activity during vocalization without auditory feedback (Fig. 5). One might predict such a smaller response because of the more limited neural activity (generated only by laryngeal somatosensory feedback or efference copy) during this condition. Importantly, speech production did vary across our four experimental conditions (Fig. 1), which may have biased our ECoG findings. We therefore address their potential effects.

Voice *F*_0_ was only slightly and non-significantly lower when vocalization occurred without feedback (Fig. 1*B*). This is atypical because the Lombard effect typically results in upward shifts in male speakers with upward or no changes from females (Junqua, 1993). There are no extant data on the effect of within-subject changes in *F*_0_ on posterior STG responses.

The largest across-experiment changes in the subject’s performance were her vocal levels as recorded by our microphone, with the largest magnitude observed during the CI microphone feedback condition (Fig. 1*D*). We speculate that this may be attributed to unequal sensitivity of the CI microphone and aux input signal paths. There was a small increase across vocalization-Aux In feedback and vocalization-no feedback conditions, which is within the range of reported effects with noise masking (Stowe and Golob, 2013) and de-activating the CI of deaf subjects (Svirsky et al., 1992). This finding is also consistent with Lombard and sidetone effects, where reductions in perceived feedback lead to increased vocal intensity produced (Chang-Yit et al., 1975; Eliades and Wang, 2012). In our subject’s case, increased vocal effort could affect cortical responses through modification of the putative efference copy and/or the concomitant changes in the magnitude of somatosensory feedback. One might initially hypothesize that such increased activity would result in larger high γ responses; however, the opposite was observed. Greater neural adaptation at higher stimulus levels may also confound interpretations of the amplitude of the high γ responses across stimulus conditions, although our datasets cannot address level-versus-response effects. Extant literature on posterior STG responses and vocal effort have not addressed this issue.

Notably, we observed that during “vocalization-playback via Aux In” and “vocalization-no auditory feedback” condition utterances, the subject produced/a/tokens at a faster rate (i.e., shorter interutterance interval; Fig. 1*C*) compared with the “vocalization-microphone feedback.” We are uncertain as to the cause of this; it may relate to subject fatigue across experimental trials. Howard et al. (2000) showed that evoked potentials from the STG are vulnerable to stimulus-rate effects, with diminished AEP’s observed as interstimulus interval was reduced from 2–0.5 s. From the “microphone feedback” to the “no feedback” condition, the subject’s median interutterance intervals were reduced from 983 to 645 ms. These values fall within the steep portion of the posterior STG ECoG amplitude-recovery function reported by Howard et al. (2000); thus, it is likely that our observed reductions in high and very high γ response are due, at least in part, to utterance-rate (i.e., neural adaption) effects.

Given this caveat regarding the differential effects of neural adaptation across our datasets, we cannot definitively ascribe the preserved γ but reduced high γ and very high γ responses during the “no feedback” condition to specific feedback mechanisms. Follow-on studies in which utterance (or stimulus) rate was controlled across experimental conditions would address this confounding effect. Our statistical analyses of the trends shown in Figure 5 suggest that, relative to γ responses, the higher-frequency ECoG components (high and very high γ responses) may be more vulnerable to utterance-rate effects. We are not aware of published data that address this possibility, although study of the influence of stimulus-rate-related neural adaptation on the different ECoG frequency responses would benefit future ECoG data interpretation and could help refine computational models of feed-forward and feed-back cortical mechanisms.

Additionally, our experiment was not designed to control for attention, which may have varied across our four experimental conditions. Clearly, self-paced vocalization requires attention to the task to produce the sounds, and indeed produced utterance duration and mean voice *F*_0_ did not statistically change across the three vocalization conditions. The playback experiment did not require a behavioral task; the subject was only instructed to listen to recorded utterances. We are unable to further quantify her attentional state during playback. We can state that she was awake and alert during all tasks, completed all tasks without difficulty, and some of her brain physiology (i.e., γ power) and behavioral (i.e., utterance duration, voice *F*_0_) metrics were similar for all conditions.

It is not possible, in this single subject and the tasks employed, to definitively separate the contribution of top-down influence and somatosensory bottom-up feedback in the preserved γ responses we obtained. Specifically, the playback condition with uncued, unpredictable, sound onset times would not be expected to produce top-down predictions that would be presumed present in the self-paced vocalization task. In addition, somatosensory bottom-up feedback is eliminated as well during playback. As mentioned, this combination raises the possibility that the unchanged γ response across all four of our experimental conditions may be because of uncontrolled attentional changes.

Changes in attentional states have been reported to modulate auditory cortical responses. ECoG responses from the posterior lateral STG are generally enhanced when subjects attended to an acoustic stimulus using a dichotic listening task (Neelon et al., 2006). In a study of ECoG responses from subjects involved in tasks where attention was directed toward a somatosensory or an auditory detection task, attention-related high γ increases occurred and were specific to somatosensory and posterior lateral STG regions, respectively (Ray et al., 2008). One study also showed modulation of scalp obtained responses during an audio-vocal integration paradigm with pitch-shifted auditory feedback (Hu et al., 2015). We hope that our unique dataset will lead to development of studies to further investigate the role of γ responses specifically, and their relationship with other frequency bands, in additional normal hearing subjects. For example, data from three human subjects reported (Fontolan et al., 2014) demonstrated such frequency segregation, using cortical depth electrodes and the Granger causality method to explore directionality of across-brain-region information flow (Granger, 1969; Bressler and Seth, 2011).

Distinct from the γ responses, we found high γ STG responses that were smaller in amplitude when vocalization occurred in the absence of auditory feedback compared with when auditory feedback was provided by the CIs. This observation supports the model where high γ responses reflect processing of auditory feedback and bottom-up processing. On the other hand, we did not observe high γ attenuation during vocalization compared with playback. While such attenuation has been reported (Flinker et al., 2010; Greenlee et al., 2011; Chang et al., 2013), posterior STG sites can show no attenuation in studies of normal hearing subjects (Flinker et al., 2010; Greenlee et al., 2011). Speech-motor-induced attenuation of activity in auditory cortex is hypothesized to be because of top-down efference mechanisms (Guenther, 2006; Tourville et al., 2008). Across-site variability in degree of STG attenuation is likely because of the varied corticocortical connectivity of different auditory subregions (Romanski et al., 1999; Romanski and Averbeck, 2009). This subject did not have electrode coverage beyond the temporal lobe (e.g., prefrontal, premotor, motor cortices) and also did not cover more posterior regions of the posterior STG, where additional speech-production activity is reported (Llorens et al., 2011; Leonard et al., 2019); thus, activity beyond the covered sites cannot be assessed.

It is characteristic of γ responses to persist for a longer duration, from stimulus onset to offset, relative to high γ responses, which decay faster from the time of stimulus onset (Crone et al., 2006, 2011). The very high γ responses were of smaller amplitude than those of γ and high γ responses. Also, relative to high γ, very high γ response durations were smaller, consistent with the pattern of shorter-duration responses as the ECoG response band increases in frequency.

### CI artifacts and the spline-Laplacian transform

As in EEG studies, we noted CI-induced electrical artifacts in ECoG recordings. At the scalp, CI artifacts can be 5–10 times larger than evoked potentials and persist across the duration of processed speech (Gilley et al., 2006). Likewise, ECoG captures both neural responses and CI artifacts. With the spline-Laplacian (Nourski et al., 2013b) revealed ECoG from this CI subject that demonstrated response characteristics like those observed in normal-hearing individuals. Our study adds to that report and provides before and after comparisons of this transforms effect on AEP and ERBP data. We identified CI artifacts were of two types: a broadband response temporally restricted to voice onset and high-frequency power increases sustained throughout the vocalization at only two sites. The more common onset artifact was observed for the two experiments in which sound stimuli were delivered to the Aux In jack but was not observed for the CI microphone experiment. The subject’s vocal output intensity did differ between these conditions (Fig. 1*D*). Thus, the CI sound processor may have reacted differently between the microphone and aux input conditions. If input levels to the processor were greater for the aux input condition, this may have caused a stronger compensatory response by the automatic gain control (AGC) circuit. As a result, a relatively large transient may have resulted before stabilization of the AGC.

The spline-Laplacian was effective at removing the onset type of CI artifact. As the algorithm computes the second spatial derivative of the neural responses, we expected a spatial sharpening of noise components since numerous recording sites contained the artifact. We indeed noted high correlations between raw and transformed waveforms. Conversely, the very focal nature of the other CI artifact type (sustained high-frequency response in only two of 96 sites) likely explains why this artifact type was not significantly altered by the transform.

It is notable that the CI artifacts were not distributed in a simple manner predicted by the proximity of recording site and CI receiver and electrodes. Several factors may be implicated. First, some electric generators of the artifacts may be dipoles of various orientations (Hughes et al., 2004). Second, in cases where the Aux In jack was used, the connecting cable may have acted as an antenna for both input signals (our feedback signals) and electric activity produced by the CI itself. Future studies in which subdural recording electrodes are used as electric sources may help shed light on the conduction of potentials across the brain.

### Comparison of cortical AEP findings with previously reported scalp AEP data

In this bilateral CI user subject, we identified AEPs from the largest amplitude cortical sites during speech production containing a dominant single peak (Fig. 5*A*, raw AEP row, sites C, D, and E). This peak had latencies of 155–250 ms and was observed, at least at site D, to be present in all four experimental conditions. At adjacent sites, the vocalization without feedback condition did not elicit an AEP response within this time window that exceeded background noise levels. Of interest, the polarity of this peak was positive during both Aux In conditions (feedback, no feedback), while the response during the Mic In and vocalization without feedback conditions were negative and slightly later compared with Aux In condition peak latencies. Because of the observed microphone level differences between the Mic and Aux In conditions (Fig. 1*D*), it is difficult to directly compare the AEPs from these two inputs and account for the morphology differences across conditions. Future work with scalp recordings where stimulus level was systematically explored could more fully characterize AEP amplitude growth and morphology. As noted earlier, our artifact-reduction transform (Fig. 5*A*, spline-Laplacian AEP row) increases waveform noise relative to the “raw” waveforms at some sites. In the case of our datasets, the raw waveforms have relatively good signal-to-noise characteristics and readily apparent neural responses, underscoring the value of examining both preprocessed and postprocessed waveforms.

Little literature reporting scalp EEG findings in CI users during speech production exists, and two studies report mismatch negativity changes without detail on waveform characteristics during produced speech (Hidalgo et al., 2019; Salo et al., 2002). Conversely, numerous reports exist for speech perception. Debener et al. (2008) obtained scalp AEPs from a single CI user, using speech stimuli and independent component analysis (ICA) to suppress stimulus artifacts and found an N1 peak at 112 ms. With dipole source modeling, the modeled generator potential at the STG exhibited a phase reversal such that a positive peak occurred at 112 ms. Sandmann et al. (2009) obtained scalp AEPs from 12 CI users, using dyadic tone combinations with 150 ms durations. Their derived “grand average” AEPs, averaged across all subjects, showed N1 at 107 ms, a relatively broad P2 with an initial peak at ∼160 ms, and the computed STG dipole waveform demonstrated a positive peak in place of the N1 peak like the Debener et al. (2008) study. Finke et al. (2016) obtained speech-evoked scalp AEPs from 13 postlingually deafened CI listeners, reporting a triphasic response with a small P1 at 65 ms, N1 at 134 ms, and a P2 peak at 240 ms; the N1 potential was generally most prominent, with peak latencies between 107 and 136 ms, somewhat shorter than what we observed in our recordings.

In conclusion, although this subject represents an extremely rare case, the data obtained offer insights into vocal motor and sensorimotor integration relevant to normal human speech control. We determined that the posterior STG produced γ, high γ, and very high γ responses not only for vocalization tasks with auditory feedback, but when the deaf subject vocalized without any auditory feedback. This finding suggests that the STG is responsive to a possible combination of attentional, efference-copy, and sensorimotor activity related to speech production. We also demonstrated that, specific to CI users, ways of reducing CI artifact contamination of neurophysiologic responses, such as in more commonly used scalp EEG, is germane to obtaining interpretable results.

## Synthesis

Reviewing Editor: Christine Portfors, Washington State University Decisions are customarily a result of the Reviewing Editor and the peer reviewers coming together and discussing their recommendations until a consensus is reached. When revisions are invited, a fact-based synthesis statement explaining their decision and outlining what is needed to prepare a revision will be listed below. The following reviewer(s) agreed to reveal their identity: NONE.

The reviewers agree that this study presents a rare opportunity to test whether STG areas activated during vocalization with auditory feedback is activated during vocalization without auditory feedback. The finding of STG activation in the absence of auditory feedback can only be explained by either efference copy or somatosensory feedback from vocalization. This is a significant finding and should be of interest to a broad scientific audience. As written however, the manuscript does not highlight the importance of the findings in either the abstract or the significance statement, making it possible readers will lose interest before they read the full manuscript. Both reviewers had additional minor comments that should be easily addressed.

Reviewer 1

This manuscript reports on a case study presenting a unique opportunity: a patient with bilateral cochlear implants (CIs) that underwent electrocorticography (ECoG) electrode array implantion for epilepsy seizure focus location. Because the ECoG array was implanted over the left temporal lobe, this case allowed the authors to investigate two questions. First, in a separate study, reported in an earlier paper, ECoG responses to externally produced auditory stimuli, including speech were examined in terms of average evoked potentials (AEPs) and event-related band-power (ERBP) spectrograms. From this the authors concluded that the patient’s responses were similar to those of normal hearing ECoG patients, which is significant considering how different the CI auditory nerve signal is from the normal cochlear signal. In the study reported here, the authors took advantage of another unique advantage of this case - that by turning off the Cis, the patient’s hearing of her own speaking could be completely blocked without the use of masking noise. This allowed them to monitor the patient’s ECoG responses in a number of speech feedback conditions: when she spoke while hearing her speech (either via the built in CI microphone or a microphone connected to the aux in port of the CIs) or spoke with the CIs turned off (no auditory feedback), or when she listened to playback of her speech via the aux in port of the CIs. They again analyzed ECoG responses in terms of AEPs and ERBPs, and used a spline-Laplacian transform to minimize CI artifacts in the recorded ECoG data. They analyzed in detail the responses at four electrodes on the STG with the largest responses across feedback conditions, focusing their analysis on evoked power in the gamma (32-70Hz), high gamma (70-150Hz), and very high gamma (156-300Hz) bands. In a subset of these electrodes, they found significant responses in all feedback conditions, including the condition where the patient spoke with no auditory feedback. They found no significant difference in responses to hearing speech feedback versus hearing playback of that speech feedback. They conclude that there are areas of the STG that respond to a combination of attention, efference copy, and sensorimotor activity occurring during speech production.

Major comments:

I think the most striking result of the study is that auditory cortex is shown to have significant responses during speaking even in the absence of auditory feedback, thus showing it must be responsive to either motor efference copy or somatosensory feedback or some combination of the two.

Unfortunately, I think this main result is not clearly spelled out in the abstract, where the authors say only “Importantly, we identified no difference in STG gamma responses between playback and vocalization with and without auditory feedback; this implicates gamma activity as relevant to attentional state.” Whereas in the conclusion of the discussion section, they are more clear, saying “We determined that the posterior STG produced gamma, high gamma, and very high gamma responses not only for vocalization tasks with auditory feedback, but when the deaf subject vocalized without any auditory feedback. This finding suggests that the STG is responsive to a possible combination of attentional, efference-copy, and sensorimotor activity related to speech production.” This is a much better statement of the study findings, and should replace the wording used in the abstract.

Authors show AEPs across ECoG grid for one feedback condition (aux in microphone, CIs turned on) but for the most part have mainly concentrated on analysis of gamma and high gamma frequency bands because of previous research showing these bands exhibit task and attentional modulations. However, there is also considerable EEG literature investigating low frequency band responses to auditory stimuli such as N1 and P2, for example. Thus, in figure 5, I would also like to see the AEPs for all these sites across all four experimental conditions, as well as lowpass filtered AEPs, to verify traditional auditory evoked waveform components like N1, P2.

In discussing why they did not find attenuated responses in the speaking with feedback conditions compared to the playback condition, the authors bring up the issue of coverage: (line 481): “Across-site variability in degree of STG attenuation is likely due to the varied cortico-cortical connectivity of different auditory sub-regions [59, 60]. This subject did not have electrode coverage beyond the temporal lobe.” More importantly, authors should note that their coverage of temporal lobe leaves out more posterior STG areas where speech production related responses have more often been found.

Minor Comments:

Spline-Laplacian transform of the EGoG data did a good job eliminating some (but not all) CI-generated artifacts, but made many channels somewhat more noisy.

At line 197, it says “Voice onset times were first determined for each trial by visual inspection of the audio waveforms.” This statement is ambiguous. Does it mean voice onset time was only subjectively determined (i.e., not by objective measure of when amplitude exceeded some threshold)? Or does it mean visual inspection was used first, followed by objective, threshold-based determination?

The differences in inter-utterance intervals and speech levels between productions made using the CI microphone and the aux in microphone suggests the patient perceived the aux in microphone signal as novel compared to the more familiar CI microphone signal. The authors say the speech level difference is consistent with the Lombard masking noise effect. It is actually more consistent with the side tone amplification effect: Chang-Yit, R., Pick, J., Herbert L., & Siegel, G. M. (1975). Reliability of sidetone amplification effect in vocal intensity. Journal Of Communication Disorders, 8(4), 317-324.

How was RMS amplitude calculated in figure 3c?

The authors justify their singling out of four electrodes for further analysis based apparently on only visual inspection of the ECoG ERBP plots in Figure 4. It would be good to have a quantitative measure that justified singling out sites B-E for further analysis.

In this analysis, the authors find no significant differences in gamma responses across all four experimental conditions. First, how were the time windows chosen for the statistical analysis? I do notice that the no vocalization aux in playback responses tend to be consistently the highest responses, though. Indeed, there is a visible trend in site D: the lower the frequency band, the greater the no vocalization aux in playback response exceeds the other responses. It argues for looking at even lower frequency bands.

Reviewer 2

Major Comments

The Abstract, in my opinion, fails to highlight the most important findings of the paper. Some, but not all, of the main findings are mentioned in the last two sentences, but there is no attempt to interpret the findings or highlight their significance, for example no mention of efference copy or of somatosensory feedback. Likewise, the Significance statement is conservative and fails to deliver the promised unique insights. I do believe that there are unique findings and insights in this paper (see above), but there is a risk that many readers will fail to learn of them without reading the rest of the paper. When the authors mention that there was no difference in STG gamma responses between playback and vocalization with and without auditory feedback, they interpret this as evidence for gamma activity being relevant to attentional state, but they don’t explain the reason for this interpretation. They also interpret the differences in high gamma responses as due to different rates of stimulus presentation and neural adaptation. Is this to argue that if these factors had been better controlled, that high gamma bands would also be unchanged across conditions? If so, this is not explained and the point is easily missed by the reader.

The authors concentrate on the differences between high gamma responses across conditions, especially the lower response magnitude during vocalization without auditory feedback. However, I am sufficiently impressed by the appearance of statistically significant high gamma responses at all under this condition. Perhaps the response magnitude is reduced due to adaptation because the interval between utterances was lower under this condition. Adaptation of high gamma responses as indeed been reported, and thus this is not particularly noteworthy. What is noteworthy, in my opinion, is that in spite of this, there is significant activation of STG without any auditory stimulus. The only thing that prevents us from interpreting this as a result of efference copy is that there is probably some sort of somatosensory, perhaps proprioceptive, feedback from vocalization, and if STG can be activated by this, we cannot attribute its activation to efference copy. Is the latter mechanism, i.e. somatosensory feedback, sufficient to explain this STG activation? Unfortunately, there was no easy way to test this in this patient since somatosensory feedback could not be eliminated. Still, I think this is an important finding, and activation of STG from either efference copy or somatosensory feedback is very interesting.

Minor Comments

I think the paper would be strengthened by including at least one time-frequency plot of the spectral responses that adequately shows responses in the different bands that were analyzed.

I think the authors should acknowledge in the Discussion that since the strongest responses occurred at the posterior edge of the grid, some activated sites may not have been recorded, and their response properties might have been different. Note that I do not believe this is a major limitation but should be acknowledged to allow for future studies that could have different findings and draw different conclusions.

In lines 192 - 193, the authors state a threshold of z = 10 for outlier reduction. How was this particular threshold chosen?

In lines 203 - 209, the ERBP extraction procedure via a Morlet wavelet decomposition is detailed. Was the log of the signal power taken prior to z-normalization?

In lines 255 - 258, it is noted that that microphone levels varied significantly across conditions. While in the discussion, it is suggested why this may have occurred, the authors may consider a statistical analysis to determine to what extent this did or did not influence the reported results, for example an ANOVA to determine whether responses within and across conditions were influenced by loudness in decibels (dB) rather than raw RMS energy (which is not log-transformed in line with human hearing).

In Figure 5, it would help to remind the reader of the timeframe from which the plots of mean band-specific power were calculated in (b).

## Author Response

Dear Dr. Portfors:

Thank you very much for your and your reviewers’ comments on our manuscript, “Neural correlates of vocal auditory feedback processing”. We have addressed all concerns raised and incorporated the additional helpful suggestions, and now submit an improved version for your consideration.

We thank you again for your time and consideration and look forward to your review of the improved manuscript.

Sincerely,

Jeremy Greenlee, MD

Professor

UIHC Department of Neurosurgery

Human Brain Research Laboratory

-

The species studied is not mentioned in the abstract. Please make sure to update both the abstract in the article file and on the submission form.

‘Human’ is now added to the abstract and submission form.

References are numbered. Please do not number the references.

We have revised the citations and references to author - date format.

The significance statement could be rewritten to more clearly highlight the importance of the findings.

We have revised the significance statement accordingly.

Reviewer 1

Major comments:

I think the most striking result of the study is that auditory cortex is shown to have significant responses during speaking even in the absence of auditory feedback, thus showing it must be responsive to either motor efference copy or somatosensory feedback or some combination of the two.

Unfortunately, I think this main result is not clearly spelled out in the abstract, where the authors say only “Importantly, we identified no difference in STG gamma responses between playback and vocalization with and without auditory feedback; this implicates gamma activity as relevant to attentional state.” Whereas in the conclusion of the discussion section, they are more clear, saying “We determined that the posterior STG produced gamma, high gamma, and very high gamma responses not only for vocalization tasks with auditory feedback, but when the deaf subject vocalized without any auditory feedback. This finding suggests that the STG is responsive to a possible combination of attentional, efference-copy, and sensorimotor activity related to speech production.” This is a much better statement of the study findings, and should replace the wording used in the abstract.

We have revised the abstract to strengthen this wording as suggested.

Authors show AEPs across ECoG grid for one feedback condition (aux in microphone, CIs turned on) but for the most part have mainly concentrated on analysis of gamma and high gamma frequency bands because of previous research showing these bands exhibit task and attentional modulations. However, there is also considerable EEG literature investigating low frequency band responses to auditory stimuli such as N1 and P2, for example. Thus, in figure 5, I would also like to see the AEPs for all these sites across all four experimental conditions, as well as lowpass filtered AEPs, to verify traditional auditory evoked waveform components like N1, P2.

We have examined the AEP waveforms in additional detail to complement our high frequency analyses. In addition to adding the requested plots to a revised figure 5, we have added context to the Discussion to compare our AEP waveforms and those from studies of subjects using cochlear-implant stimulation with scalp recording electrodes. While there are some differences across our study and the scalp-recording studies, the AEPs do occur over comparable, overlapping, post-stimulus time intervals.

In discussing why they did not find attenuated responses in the speaking with feedback conditions compared to the playback condition, the authors bring up the issue of coverage: (line 481): “Across-site variability in degree of STG attenuation is likely due to the varied cortico-cortical connectivity of different auditory sub-regions [59, 60]. This subject did not have electrode coverage beyond the temporal lobe.” More importantly, authors should note that their coverage of temporal lobe leaves out more posterior STG areas where speech production related responses have more often been found.

This is an important caveat to our recording locations, and we have added text to the discussion to note this limitation with appropriate references.

Minor Comments:

Spline-Laplacian transform of the EGoG data did a good job eliminating some (but not all) CI-generated artifacts, but made many channels somewhat more noisy.

We have added text to the Discussion section specifically noting this fact, along with the value of presenting both pre- and post-processed AEP waveforms (see Fig 5).

At line 197, it says “Voice onset times were first determined for each trial by visual inspection of the audio waveforms.” This statement is ambiguous. Does it mean voice onset time was only subjectively determined (i.e., not by objective measure of when amplitude exceeded some threshold)? Or does it mean visual inspection was used first, followed by objective, threshold-based determination?

This sentence was not clearly written. We have clarified this text to reflect our technique of automated threshold detection of each vocal onset in the audio tract and confirmed with visual inspection.

The differences in inter-utterance intervals and speech levels between productions made using the CI microphone and the aux in microphone suggests the patient perceived the aux in microphone signal as novel compared to the more familiar CI microphone signal. The authors say the speech level difference is consistent with the Lombard masking noise effect. It is actually more consistent with the side tone amplification effect: Chang-Yit, R., Pick, J., Herbert L., & Siegel, G. M. (1975). Reliability of sidetone amplification effect in vocal intensity. Journal Of Communication Disorders, 8(4), 317-324.

Thank you for this reference and insight. We have added this reference and point to the discussion.

How was RMS amplitude calculated in figure 3c?

This was calculated using the rms function in the Matlab signal processing toolbox.

The authors justify their singling out of four electrodes for further analysis based apparently on only visual inspection of the ECoG ERBP plots in Figure 4. It would be good to have a quantitative measure that justified singling out sites B-E for further analysis.

The reviewer is correct that we selected sites B-E simply from visual inspection of the broadband spectrogram responses from all 96 channels (e.g. Fig. 4). Since this was the only subject in the study and we therefore did not need to identify / match / control for multiple different anatomical brain recording locations across multiple subjects, we felt the clear, notably larger and visualized amplitude responses from these sites was sufficient to justify not further analyzing other electrodes for this report.

In this analysis, the authors find no significant differences in gamma responses across all four experimental conditions. First, how were the time windows chosen for the statistical analysis? I do notice that the no vocalization aux in playback responses tend to be consistently the highest responses, though. Indeed, there is a visible trend in site D: the lower the frequency band, the greater the no vocalization aux in playback response exceeds the other responses. It argues for looking at even lower frequency bands.

Thank for your suggestion. We utilized a pre-voice onset time window (-390 to -100 ms) for baseline activity and a post-onset window (0 - 400 ms) for statistical comparisons. The pre-onset window was selected to fall in a ‘silent’ period and avoid contamination from any preceding utterance, and also allow for identification of any activity occurring prior to vocalization. The post-onset window was chosen to cover the time period of maximal high-frequency response amplitude. We have added this text to the methods. As noted above to a prior reviewer comment, we have revised figure 5 and include the AEP analyses (see above critique response) to provide additional information on low frequency responses.

Reviewer 2

Major Comments

The Abstract, in my opinion, fails to highlight the most important findings of the paper. Some, but not all, of the main findings are mentioned in the last two sentences, but there is no attempt to interpret the findings or highlight their significance, for example no mention of efference copy or of somatosensory feedback. Likewise, the Significance statement is conservative and fails to deliver the promised unique insights. I do believe that there are unique findings and insights in this paper (see above), but there is a risk that many readers will fail to learn of them without reading the rest of the paper. When the authors mention that there was no difference in STG gamma responses between playback and vocalization with and without auditory feedback, they interpret this as evidence for gamma activity being relevant to attentional state, but they don’t explain the reason for this interpretation. They also interpret the differences in high gamma responses as due to different rates of stimulus presentation and neural adaptation. Is this to argue that if these factors had been better controlled, that high gamma bands would also be unchanged across conditions? If so, this is not explained and the point is easily missed by the reader.

Thank you for your suggestion. This was noted also by the reviewer 1 (see above) and we have revised the wording of the abstract to highlight these points better.

The authors concentrate on the differences between high gamma responses across conditions, especially the lower response magnitude during vocalization without auditory feedback. However, I am sufficiently impressed by the appearance of statistically significant high gamma responses at all under this condition. Perhaps the response magnitude is reduced due to adaptation because the interval between utterances was lower under this condition. Adaptation of high gamma responses as indeed been reported, and thus this is not particularly noteworthy. What is noteworthy, in my opinion, is that in spite of this, there is significant activation of STG without any auditory stimulus. The only thing that prevents us from interpreting this as a result of efference copy is that there is probably some sort of somatosensory, perhaps proprioceptive, feedback from vocalization, and if STG can be activated by this, we cannot attribute its activation to efference copy. Is the latter mechanism, i.e. somatosensory feedback, sufficient to explain this STG activation? Unfortunately, there was no easy way to test this in this patient since somatosensory feedback could not be eliminated. Still, I think this is an important finding, and activation of STG from either efference copy or somatosensory feedback is very interesting.

Thank you for your insights. We agree that this pattern of STG responses observed in this patient are very intriguing for the reasons you outline. In hindsight, a powerful additional experiment in this subject would have been to use topical anesthetics in the mouth / larynx to try and reduce / eliminate somatosensory feedback and look at any resultant changes in gamma / high gamma responses.

Minor Comments

I think the paper would be strengthened by including at least one time-frequency plot of the spectral responses that adequately shows responses in the different bands that were analyzed.

We have included spectrogram plots for sites A-E to show time-frequency responses from 1-300 Hz for these sites (Fig. 2b).

I think the authors should acknowledge in the Discussion that since the strongest responses occurred at the posterior edge of the grid, some activated sites may not have been recorded, and their response properties might have been different. Note that I do not believe this is a major limitation but should be acknowledged to allow for future studies that could have different findings and draw different conclusions.

We have added additional comments to the discussion to reflect this coverage limitation.

In lines 192 - 193, the authors state a threshold of z = 10 for outlier reduction. How was this particular threshold chosen?

We did arbitrarily use z = 10 as our threshold to exclude trials containing high amplitude voltage deflections deemed artifactual. In addition, all trials were visually inspected for artifacts.

In lines 203 - 209, the ERBP extraction procedure via a Morlet wavelet decomposition is detailed. Was the log of the signal power taken prior to z-normalization?

Log was taken after normalization to baseline power.

In lines 255 - 258, it is noted that that microphone levels varied significantly across conditions. While in the discussion, it is suggested why this may have occurred, the authors may consider a statistical analysis to determine to what extent this did or did not influence the reported results, for example an ANOVA to determine whether responses within and across conditions were influenced by loudness in decibels (dB) rather than raw RMS energy (which is not log-transformed in line with human hearing).

This is an excellent point about stimulus level effects and their possible influence on our results. We have addressed this issue in the Revision. An adequate examination of stimulus level effects are complex for at least two reasons. First, to address level effects, we would need to know the “operating point” of the cortical neural system relative to level and that would require access to a stimulus-level vs. cortical output function for each stimulus condition. Such information would help us determine, among other things, the degree to which neural adaptation may interact with level. We do not have such data sets and therefore believe that further analysis and comment would be highly speculative. Furthermore, CI’s strongly compress the dynamic range of speech into a range of less than 20 dB (e.g., Galvin and Fu 2009) such that the decibel scale seen at a microphone output does not map simply into human perceptual dynamic ranges. For these considerations, we feel that acknowledgement of these factors is appropriate, while conducting an ANOVA likely goes beyond the scope of this paper’s data sets.

Galvin J.J.III, Fu Q-J. 2009. Influence of stimulation rate and loudness growth on modulation detection and intensity discrimination in cochlear implant users. Hear Res 250, 46-54. PMID: 19450432

In Figure 5, it would help to remind the reader of the timeframe from which the plots of mean band-specific power were calculated in (b).

We have added a title to the figure to indicate this.
